# Case report: reverse lateral arm flap in a patient with previously harvested radial artery

**DOI:** 10.1080/23320885.2022.2099395

**Published:** 2022-07-19

**Authors:** Zahir T. Fadel, Mohammed B. Ashi, Weaam S. Magram

**Affiliations:** a Division of Plastic Surgery, Department of Surgery, Faculty of Medicine, King Abdulaziz University, Jeddah, Saudi Arabia; b Department of Plastic and Reconstructive Surgery, National Guard Hospital, Jeddah, Saudi Arabia

**Keywords:** Elbow reconstruction, flaps, olecranon wounds, posterior elbow wounds, upper extremity flaps

## Abstract

The radial artery supplies various locoregional flaps used for elbow reconstruction. A reverse lateral arm flap is a reliable choice, despite sacrificing the radial artery in some cases. We describe using a reverse lateral arm flap for elbow soft tissue reconstruction in a patient with a previously harvested radial artery.

## Introduction

The elbow is a highly functional area with multiple considerations to keep in mind when planning for reconstruction [1,2]. The risk of developing stiffness is high, particularly when radiation is a factor. Therefore, providing a thin and reliable fasciocutaneous flap is preferred to facilitate early mobilization. Common regional flaps for elbow reconstruction are the radial forearm flap and reverse lateral arm flap (RLAF).

The radial artery supplies many of the locoregional flaps around the elbow. Thus, eliminating the radial artery can limit the available reconstructive options. The RLAF receives its blood supply *via* the recurrent radial artery (RRA), in addition to the interosseous recurrent artery (IRA) [3–5]. In this report, we describe the use of RLAF for elbow soft tissue reconstruction in a patient with a previously harvested radial artery. All procedures followed were in accordance with the ethical standards of the responsible committee on human experimentation (institutional and national) and with the Helsinki Declaration of 1975, as revised in 2008. Informed consent was obtained from the patient included in the study.

## Case presentation

**Figure 1.**
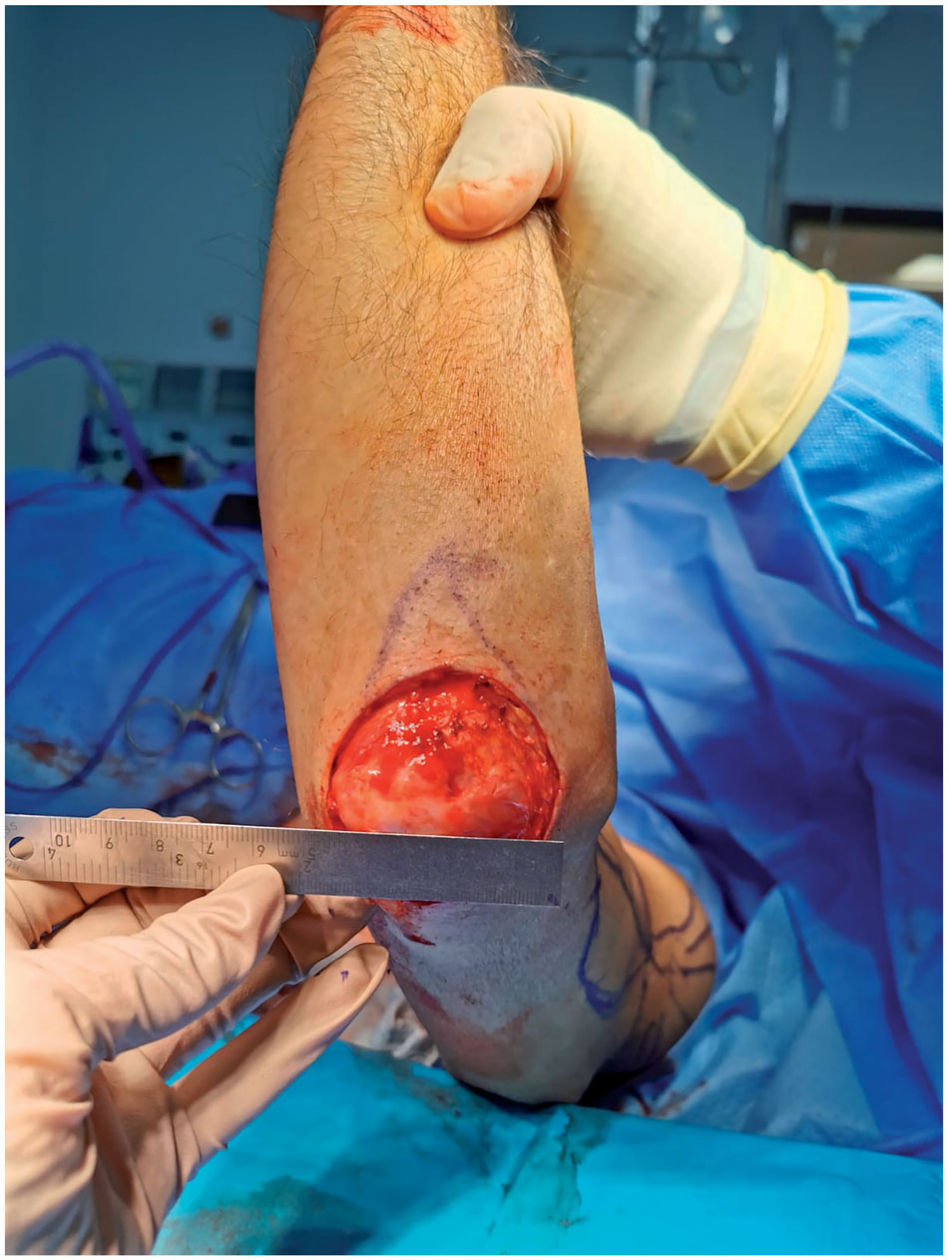
Left elbow defect following wide local excision of a Merkel cell carcinoma lesion.

A 70-year-old male with left elbow Merkel cell carcinoma. A volar forearm scar was identified starting at the cubital fossa, related to harvesting the radial artery for coronary artery bypass. There was no distant metastasis identified in his work-up. Wide local excision was performed, with a 2 cm margin. Sentinel lymph node biopsy was positive; therefore, level I-II axillary lymph node dissection was completed. The resulting defect measured 5 × 5 cm over the olecranon (Figure 1). RLAF was planned for reconstruction.

### Flap harvest

**Figure 2.**
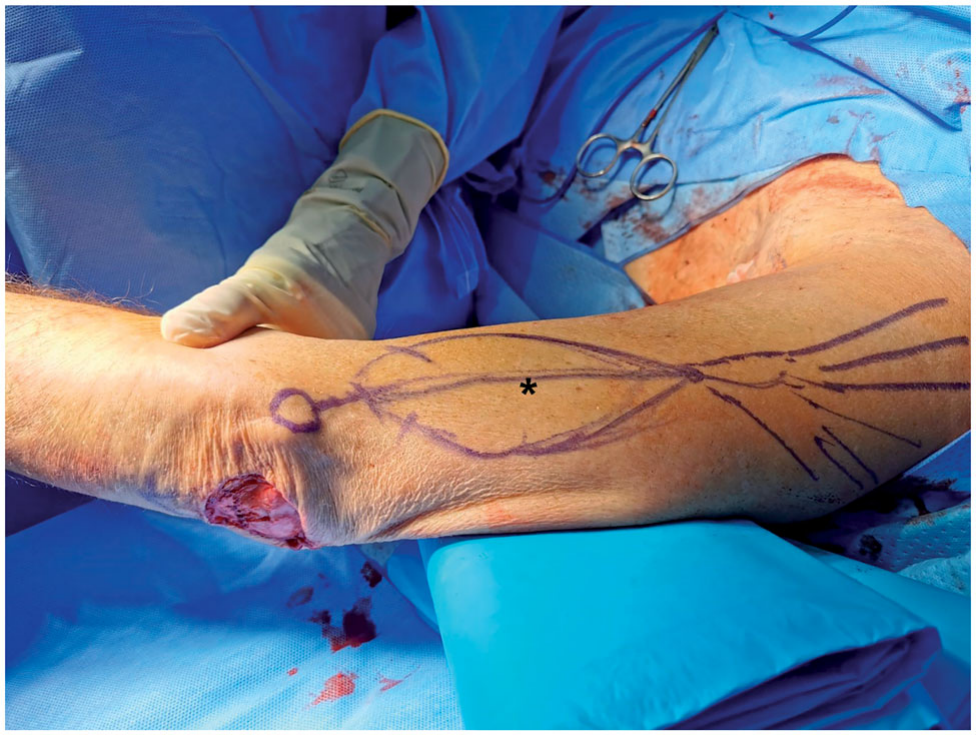
Reverse lateral arm flap design along the axis of the lateral intermuscular septum, extending from the deltoid insertion to the lateral epicondyle. (*) Audible doppler signal of the targeted perforating vessels.

The axis of the lateral intermuscular septum was marked, from the deltoid insertion to the lateral epicondyle. The skin island design was centered over the lower third, measuring 15 × 8 cm (Figure 2). A hand-held doppler was used to confirm the location of perforating vessels.

**Figure 3.**
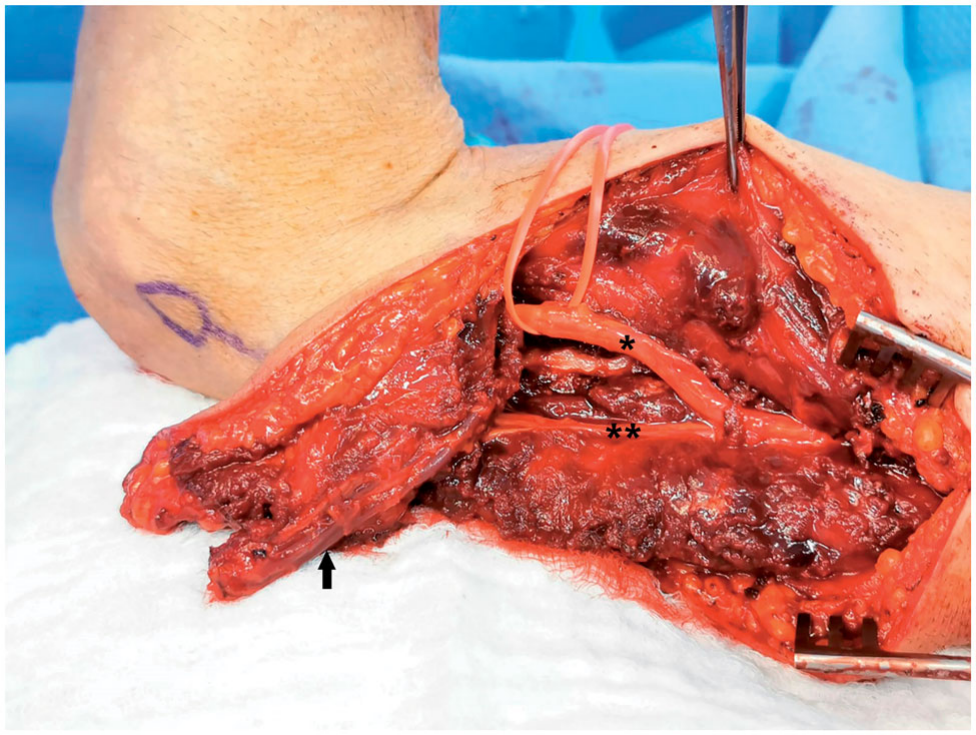
Reverse lateral arm flap pedicle dissection (Arrow) and sparing of the radial nerve (*) and posterior cutaneous nerve of the arm (**).

The posterior edge of the flap was incised and the dissection was carried down to the sub-fascial plane towards the lateral intermuscular septum. Two septocutaneous perforators were identified and preserved. The pedicle was dissected free from the surrounding tissue and nerves, protecting the radial nerve and posterior cutaneous nerve of the arm (Figure 3). The proximal end of the radial collateral artery (RCA) was ligated, leaving an adequate length for potential supercharge if necessary.

**Figure 4.**
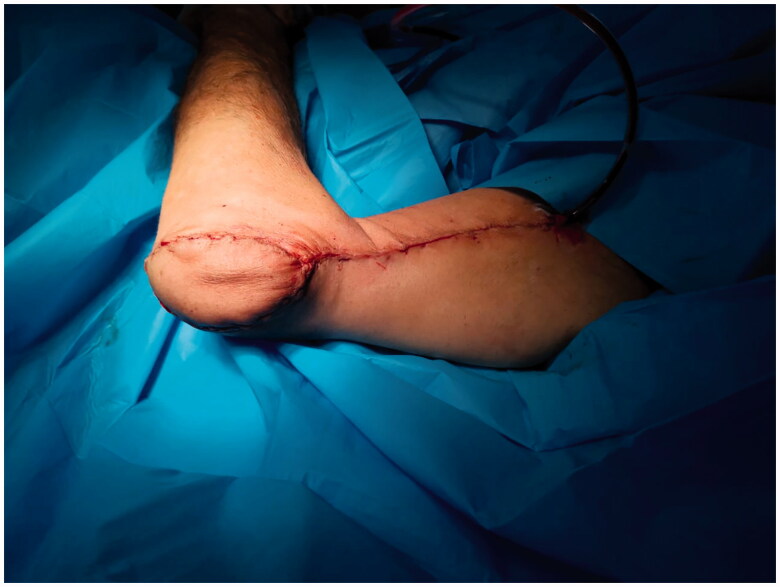
Reverse lateral arm flap rotation and inset.

The flap was rotated 180 degrees and inset to the defect. The flap perfusion was found to be adequate. The overlying skin bridge was divided to avoid compression on the pedicle and the donor site was closed primarily without tension (Figure 4).

### Post operative course

**Figure 5.**
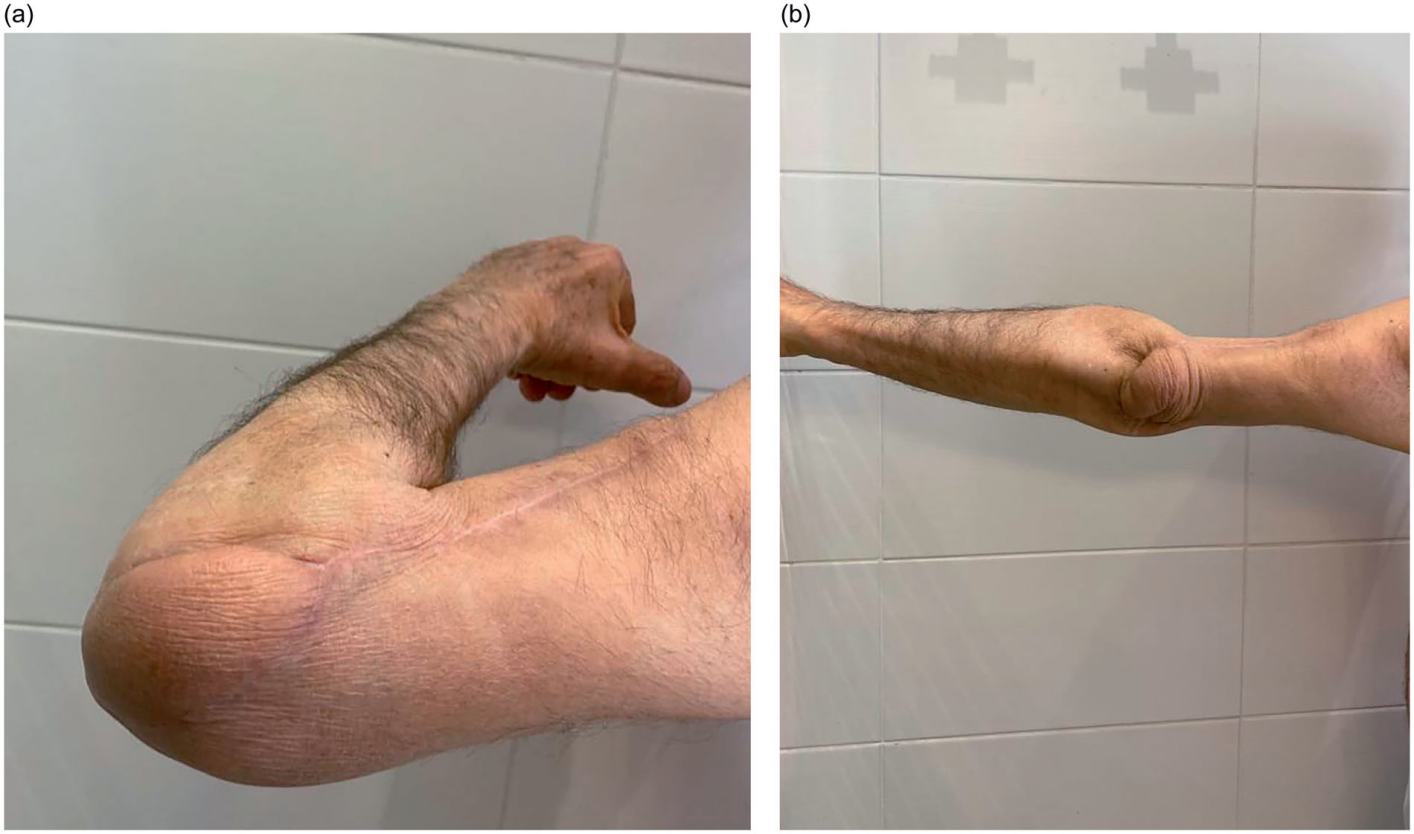
Recovery of elbow range of motion was shown at six months follow-up. (a) Full flexion (b) Full extension.

The flap viability was maintained, and wounds were managed until complete healing. The patient eventually had to receive radiotherapy. Recovery of full elbow range of motion was achieved six months post-surgery with physiotherapy (Figure 5).

## Anatomy

Maruyama and Takeuchi [3] described the RLAF as a modification of fasciocutaneous flaps previously reported in the literature [6,7]. The RCA, a terminal branch off the profunda brachii artery, bifurcates at the level of the brachioradialis origin into the anterior and posterior radial collateral arteries (ARCA & PRCA) [8]. The PRCA travels within this septum and communicates with the IRA. The ARCA passes anterior to the lateral intermuscular septum communicating with the RRA [3]. This artery commonly takes off proximally, which should be preserved during the harvest of the radial artery for coronary artery bypass [9]. Hamahata et al. described four different origins for the RRA, including the radial artery proper, root of the radial, brachial, or ulnar artery [10]. The RRA is often reported as the main pedicle of the RLAF [3]. However, Culbertson and Mutimer described the PRCA-IRA being the main pedicle in their paper [4].

Combining the above descriptions of vascular supply around the elbow is referred to as the olecranon plexus. Wei and Mardini described this plexus as being formed by the communication of PRCA, ARCA, IRA, RRA, ulnar recurrent, and the inferior ulnar collateral [5]. The RLAF receives its blood supply through the olecranon plexus [3–5]. It is important to protect this vascular plexus and avoid dissecting the RRA or IRA, but rather leave a 2 cm fasciocutaneous base [5].

## Discussion

The elbow is a highly functional joint, requiring thin, durable, and stable soft tissue that facilitates early mobilization. Providing a well vascularized reliable flap is essential, particularly when radiotherapy is required. Many available locoregional flaps are based on the radial artery or its branches, such as the radial forearm and regional muscle flaps. The patient presented had a history of radial artery harvest for coronary artery bypass. This precludes the ability to use forearm fasciocutaneous or muscle flaps purely based on the radial artery. Additionally, it may compromise proximal regional flaps without careful planning.

Considering the advantages of locoregional fasciocutaneous flaps, we elected to use the RLAF. Septocutaneous perforators identified in our case were proximal, precluding a propeller-type lateral arm flap [11]. Since the radial artery was sacrificed, the possibility of the blood supply to the flap being compromised was kept in mind. We were prepared to anastomose the RCA to the proximal stump of the radial artery as a supercharge if needed. However, flap perfusion following inset was adequate without requiring any further intervention. Flap advancement was somewhat limited by the skin bridge near the pivot point. We preferred to incise the skin superficially in order to facilitate transposition without causing compression on the pedicle. We did not experience significant venous congestion, which can be a concern in some cases [11].

Different flaps are described for elbow defects, including random, axial muscle or fasciocutaneous flaps. Reconstructive goals and surgeon expertise should be taken into consideration while planning reconstruction. Examples of fasciocutaneous flaps include the radial forearm, ulnar forearm, posterior interosseus artery, and propeller flaps. Muscle flaps such as flexor carpi ulnaris and anconeus can be used in certain cases [1,2]. RLAF is a reliable option for elbow defects, particularly when radiation is necessary. It provides consistent anatomy and relatively straightforward dissection, with minimal donor site morbidity. The radial forearm flap remains the most preferred option by many surgeons. However, in cases where the radial artery is sacrificed, RLAF can be an excellent alternative to consider. Performing careful doppler assessment or computed tomography angiography is important for accurate planning. Expertise in microsurgery is required to be able to perform a supercharge anastomosis, if necessary.

## Conclusion

In summary, elbow soft tissue defects can be challenging to reconstruct. The radial artery supplies many of the locoregional flaps. Without adequate planning, eliminating the radial artery can limit the available options. The RLAF can be a reliable and favorable choice, despite the absence of the radial artery in some cases.

## Informed consent

Informed consent was obtained from the patient of the study.
